# Skiing Injuries at the Dizin Ski Resort

**DOI:** 10.5812/traumamon.3799

**Published:** 2012-05-26

**Authors:** Amir Hossein Khalilifar, Mohammad Hassan Kazemi, Arya Hamedanchi, Mohammad Javad Hosseini

**Affiliations:** 1 Iran Triathlon Federation, Tehran, IR Iran; 2 NEZAJA Preventive Medicine Department, Tehran, IR Iran; 3 University of Social Welfare and Rehabilitation Sciences, Tehran, IR Iran; 4 Molecular Biology Research Center, Baqiyatallah University of Medical Sciences, Tehran, IR Iran

**Keywords:** Skiing, Wounds and Injuries, Dizin Ski Resort

## Abstract

Skiing is one of the more popular winter sports which may cause injuries. The objective of this study was to identify the most common types of injuries in Iran’s largest ski resort. This cross-sectional descriptive study was performed on 1233 of patients admitted to the Dizin Resort Infirmary in 2008-2009. Obtained data included age, gender, injury type and medical interventions. All data were analyzed by SPSS 16.0 software.

Results showed that 75% of the patients were male and 25% female. The mean age was found to be 27.86 (± 9.95) years. Most patients were between 20-29 years old (55.2%). The most common injury was knee trauma (14.4%). Other common injuries were soft tissue injury (12.1%), shoulder trauma (8.1%), head and face trauma (7%) and wrist trauma (5.5%) respectively. There was a significant relationship between age and sex i.e. the age in women was less than men’s (P < 0.001). We found a relationship between age and injury type. The lowest mean age (24.83) was reported in the patients with head and face injuries and the highest mean age (44.5) was in the patients with malleolus fracture (P < 0.001). Additionally, sex and knee trauma proved to be connected with more prevalence in women (P = 0.001). There was also a significant relationship between sex and shoulder injuries showing a higher prevalence in men (P = 0.015).

## 1. Background

The history of skiing dates back thousands of years. The oldest ski was found in Russia, dating back 5000- 6300 BC (1). Millions of people in the world go skiing every year. Physical activities are important parts of life in modern societies, and injuries inevitably occur in physical exercises and competitions (2). Skiing may cause injuries in athletes as well. Some risk factors in skiing include: High speed, hitting other skiers or obstacles, trauma caused by ski equipment to the body or to others and sudden movements which cause muscular strain or tremendous pressure on the joints (3, 4).

The Dizin Ski Resort is the first ski resort in Iran to hold formal competitions, approved by the International Ski Federation. Dizin is located in the northern mountains of Tehran in Gajereh. The lowest part of Dizin is 2650 m and the highest part is 3600 m above sea level. The ski season in Dizin is longer than other resorts in Iran starting in November and ending in May (5).

## 2. Objectives

The objective of this study was to identify the most common injuries in the largest ski resort in Iran. Given that no similar study has been conducted in Iran yet, the information can help physicians as well as rescue teams to provide better services for injured skiers.

## 3. Materials and Methods

This study assessed 1223 patients during the ski season of 2008-2009. In this research, data were recorded by paramedics or physicians at the Dizin Infirmary. All practicing physicians were orthopedic Surgeons or GPs who had passed the international course on ski medicine. Patients with unspecified complaints were excluded from the study. The data included: age, sex, chief compliant or injury types and medical interventions. All data were analyzed by SPSS16 using Kolmogrov-Smirnov, Mann-Whitney, Kruscal-Wallis, Chi-Square and Fisher Exact tests.

## 4. Results

Males comprised 75% of the cases and 25% of the cases were females. The mean age was 27.86 (± 9.5). The youngest patient was 6 years-old and the oldest was 73 years-old; 1.1% of the patients were younger than 10 years-old; 13% were between 10-19 years old; 55.2% were 20 to 29; 16.7% of patients were between 30 and 39 years-old; 8.4% of patients were between 40 to 49 years-old; 5.2% of patients were between 50 to 59 years-old ;0.2% of patients were between 60 to 69 and 0.2% of patients were above 70 years-old.

The largest group ranged between 20-29 years-old (679 patients) and the smallest groups were people between 60-69 years and above (2 patients). The most common chief complaint among the patients of the emergency room was their medical condition they had (33.8%). The most frequent injury was knee trauma (14.4%). Respectively, soft tissue injury (12.1%), shoulder injury (8.1%) and head and neck trauma (7%) were the 3rd to 5th common causes for referring to the emergency room; 33.1% of the patients received only an analgesic or anti-inflammatory drug. Bandage or splint was used for 22.7% of the patients; 9.6% of the patients received other kinds of drugs; in 9.2% of the cases no treatment was needed.

There was a significant relationship between sex and age, indicating that the mean age in females is lower than the mean age in males (P < 0.001). The results also indicated a relationship between age and the type of trauma (P < 0.001). The lowest mean age was 24.83 years which was seen in the patients with head and neck trauma and the highest mean age was 44.5 years which was seen in the patients with ankle fracture (Table 1).

**Table 1.** Mean age in different types of causes for referral.

Causes	Mean age	Number of patients	SD
Medical conditions	31.12	394	10.997
Toe trauma	25.89	9	9.943
Ankle trauma	25.26	43	5.113
Shin trauma	25.17	35	6.586
Knee trauma	26.25	167	7.992
Thigh trauma	29	8	11.032
Pelvic trauma	25.75	4	4.031
Abdominal trauma	25	5	3.464
Backbone trauma	26.41	17	8.375
Chest trauma	25.33	9	8
Shoulder trauma	26.06	95	7.537
Arm Trauma	25.62	13	9.403
Elbow trauma	26.5	30	10.281
Forearm trauma	27.58	12	9.405
Wrist trauma	24.84	64	9.585
Finger trauma	28.88	26	11.19
Neck trauma	30.09	11	8.86
Head & face trauma	24.83	82	9.388
Ankle fracture	44.5	2	3.536
Soft tissue trauma	27.3	142	10.322
Total	27.9	1148	9.893

Based on the results, there was a significant relationship between knee trauma and sex. Specifically knee trauma was more common in women compared to men (P < 0.001). Moreover, there was also a significant relationship between sex and shoulder trauma which was statistically more common in men (P = 0.015, Table 2).

**Table 2.** Causes for referral of males and females.

Type of injury	Female	Male
Medical conditions	74	320
Toe trauma	3	6
Ankle trauma	14	29
Shin trauma	10	24
Knee trauma	61	107
Thigh trauma	4	4
Pelvic trauma	2	2
Abdominal trauma	3	2
Backbone trauma	5	12
Chest trauma	1	8
Shoulder trauma	14	81
Arm Trauma	7	6
Elbow trauma	8	22
Forearm trauma	2	9
Wrist trauma	20	44
Finger trauma	6	20
Neck trauma	7	4
Head & face trauma	23	59
Ankle fracture	1	1
Soft tissue trauma	28	114
Total	293	874

## 5. Discussion

Based on the results of our study, the most common trauma was respectively: knee trauma, soft tissue trauma, shoulder trauma, head and neck trauma and wrist trauma. Longrom *et al*. in Scotland conducted a case-control study in ski season of 1999-2000 on 674 injured skiers and 336 non-injured persons; 58.9% of injured patients were male and 41.1% were female. Unlike our findings, there was no significant relationship between sex and injury. The most common injuries were in the age group of younger than 16 years. In addition, age had a significant relationship with injury (P = 0.001); 12.5% of the injured had fractures, 51.7% had sprains, 9.6% had lacerations, 2.7% had joint dislocation, and 23.5% other injuries (6). Bridges et al. in eastern Colorado conducted a case series study on 1332 injured skiers. The mean age was 29.3 (±17.2). In that study, the incidence of injury in men was higher than that of women (OR=1.061). Additionally, the most common injury among skiers was knee trauma which matches our findings (7).

As some types of injuries occur in a specific sex or age group, safety precautions are more important for them. For example, using safety helmets can play an important role in prevention of trauma in the lower ages and safety precautions for prevention of ankle fracture in the older skiers are important. It is also recommended to use knee supports to prevent trauma in women. Having considered the frequency of injuries caused by skiing, more emphasis on the preventive methods is essential. Providing adequate training by ski instructors and an emphasis on warming up before skiing can help reduce such injuries.
